# Distribution of out of pocket health expenditures in a sub-Saharan Africa country: evidence from the national survey of household standard of living, Côte d’Ivoire

**DOI:** 10.1186/s13104-019-4048-z

**Published:** 2019-01-15

**Authors:** Akissi Régine Attia-Konan, Agbaya Stéphane Serge Oga, Amadou Touré, Kouakou Luc Kouadio

**Affiliations:** 1 Department of Public Health, Hydrology and Toxicology, UFR Pharmaceutical and Biological Sciences, University of Félix Houphouet Boigny, BP V34 Abidjan, Côte d’Ivoire; 2 National Institute of Statistic of Côte D’Ivoire, Abidjan, Côte d’Ivoire

**Keywords:** Out of pocket expenditure, Direct payment, Health expenditure, Côte d’Ivoire

## Abstract

**Objective:**

The purpose and objective of our research is to identify the determinants of the out of pocket (OOP) health expenditures in the population of Ivory Coast and the ratios across three different area; Abidjan, the rural and urban area. We used data from the 2015 standard households living survey conducted by the National Institute of Statistic.

**Results:**

About 6315 (13.3%) of the participants had experienced OOP health expenditure. There was significant differences in the self-reported OOP between these three areas (p < 0.001). The overall mean of OOP expenditure among all participants was 16,034.33 XOF (29 USD). People in Abidjan spent an average of 1.6 and 1.5 times more than those in the rural and urban areas respectively (p < 0.001). Hospitalization is the highest expenditure item in terms of money spent, while drugs are the most common item of expenditure in terms of frequency, regardless of the place of residence. Female gender, high social economic status and large household size increase OOP health expenditure significantly in all areas of residence when insurance reduce it. To reduce the impact of the direct payments there is a need to take into account social demographic factors in addition to economic factor in health policy development.

**Electronic supplementary material:**

The online version of this article (10.1186/s13104-019-4048-z) contains supplementary material, which is available to authorized users.

## Introduction

In the low and middle income countries, health expenditures are financed mainly by households through direct payments [1, 2]. These direct payments constitute a financial risk to households using health services [1, 3] and expose households to a catastrophic situation that can lead to impoverishment [4–6]. In fact, households, especially the poor ones, will abandon treatments or borrow money to pay when treatment costs become too high [5]. Therefore, the goal of sustainable development project is achieving a significant reduction of these direct payments [7].

About 808 million people were exposed to catastrophic health expenditures in 2017, over 80% of whom are from Asia and Africa [8]. The predictors of direct payments were mainly economic and demographic, such as the household size, the existence of health insurance [9, 10], and also the existence of a chronic illness in the household [11]. In Côte d'Ivoire, direct payments accounted for 32.55% of current health expenditure in 2015 [12]. They were introduced in 1987 by the Bamako Initiative, which instituted community participation and cost recovery [13]. Payment exemption models for a particular group of the population and mutual health insurance schemes have been developed to reduce these payments, but these mechanisms have proved to be ineffective [14].

The distribution of direct payments according to socio-demographic characteristics and place of residence has rarely been studied in West Africa countries, although rural households have lower amount spent on health expenses and are often vulnerable [15–17]. Since urbanization contributes to an increase in health expenditure [18], it is important to identify the weight of this urbanization for better health policy development.

The aim of our study was to estimate the direct payments made to take care of health expenditures and their distribution according to different areas of residence.

## Main text

### Methods

This study uses data from the standard households living cross-sectional survey conducted from 23 January to 25 March 2015 by the National Institute of Statistics of Côte d'Ivoire (NIS). The NIS employed a stratified 2-degree random sampling by using the 2014 General Population and Housing Census results as a sampling frame. The final sample included 12,899 households and 47,635 participants. The details of the sampling participants’ information are available elsewhere [19]. Trained fields staff conducted house to house interviews using structured questionnaires [19]. At the individual level, the data included demographic, socioeconomic characteristics and self-reported out-of-pocket (OOP) health expenditures during the last 3 months. Access to safe drinking water, toilet facilities, electricity, as a source of lighting, and gas, as an energy source, were extracted. The OOP health expenditures were estimated from utilization of modern facilities such as public and private curative or preventive health services, over-the-counter medications and use of traditional healer services.

OOP health expenditure resulting from the use of health goods and services was response variable. The unit of analyzes is individual’s OOP expenditure. The purpose of these payments was also considered as expense item. The factors determining the health care demand according to Anderson’s conceptual framework were used as independent variables [20]. We included gender, age and marital status as predisposing factors. Level of education, assistance with the payment of health care expenses (insurance policy or financial aid), household’s size and a convenience score as proxy measures for wealth were considered as enabling factors. The financial aid consisted in support from a relative or local/national authorities sometimes granted on request. The convenience score was calculated using five variables which provided data on type of water supply, sanitation, disposal of household waste, source of lighting and energy. The self-reported chronic morbidity such as diabetes, cardiovascular illness was included as need factors. The dependent variable OOP health expenditure was recorded in West Africa CFA currency (XOF) (1 USD = 560,250 XOF) [21].

Initially, we analyzed the study variables using descriptive statistics. Because of positively skewed OOP health expenditure data, the log of this variable was computed to better approximate a normal distribution for inference. The associations between the log of OOP health expenditure and the independent variables into each residence area on one hand and the three residence areas into each modality of the independent variables on the over hand were explored using analysis of variance (Anova) or t-test (as appropriate). The three residence areas are Abidjan, the economic capital, the urban and rural area. Generalized estimating equation (GEE) was used to modeling response variable because of possible unknown correlation between outcomes [22]. More than one person in a household could incur expenditure and health-seeking decision within household is probably correlated. To assess pattern of OOP health expenditure, we used a generalized linear regression model (GLM). Data were analyzed using statistical software STATA 12.0. A p value of less than 0.05 was considered significant.

### Results

About 6315 (13.3%) of the 47,635 participants had experienced OOP health expenditure within the last 3 months. The self-reported OOP expenditure differed significantly between the three areas of residence (p < 0.001). People living in Abidjan has reported more frequently OOP expenditure: Rural 3311 (12.5%), Urban 2296 (13.9%) and Abidjan 708 (15.2%). The overall median of OOP expenditure among all participants was 10,000 XOF (17.85 USD) and the mean was 16,034.33 XOF ± 32,951.4 (28.62 USD ± 58.82). People in Abidjan spent an average of 1.6 and 1.5 times (23,447.7 XOF ± 55,136.1) (41.85 USD ± 98.41) more than in rural and urban respectively (p < 0.001).

Table 1 shows that OOP expenditure was significantly higher, in terms of frequency and amount, in Abidjan than in urban and rural areas in all expense items except consultation in traditional healers and traditional drugs.

**Table 1:** Estimation of direct payment (XOF) by expense item according to the area of residence (n = 47,635)

Expense item	Rural (26,488)		Urban (16,477)		Abidjan (4670)		p value^a^
	n (%)	Mean ± SD (median/min–max)	n (%)	Mean ± SD (median/min–max)	n (%)	Mean ± SD (median/min–max)	
Hospitalisation	150 (0.57)	39,150.0 ± 74,761.9 (18,000/200–600,000)	113 (0.69)	40,472.1 ± 102,317.1 (12,000/500–856,000)	57 (1.22)	89,018.0 ± 145,088.1 (30,000/1000–800,000)	0.003*
Drugs	3100 (11.70)	10,752.8 ± 8572 (8000/100–53,000)	2191 (13.30)	11,608.4 ± 9518.8 (9000/100–60,000)	683 (14.63)	12,729.9 ± 9708.4 (10,000/100–47,000)	< 0.001*
Laboratory/radiology	321 (1.21)	7855.9 ± 13,381.6 (3000/100–100,000)	217 (1.32)	12,903.8 ± 36,464.8 (5000/300–350,000)	91 (1.95)	21,853.9 ± 47,314.3 (8000/100–400,000)	< 0.001*
Consultation modern health services	1032 (3.90)	1222.5 ± 6297.2 (1000/100–200,000)	909 (5.52)	1275.6 ± 1501.0 (1000/50–28,000)	278 (5.95)	2114.9 ± 1483.9 (2000/300–5000)	< 0.001*
Consultation traditional medicine	126 (0.48)	7717.8 ± 21,663.24 (2000/105–200,000)	64 (0.39)	5300.1 ± 11,277.0 (2000/105–80,000)	20 (0.43)	9490.3 ± 14,482.4 (1750/500–50,000)	0.3751
Traditional drugs	620 (2.34)	6251.4 ± 23,154.0 (2000/100–500,000)	358 (2.17)	6311.6 ± 28,089.5 (1500/100–45,0000)	89 (1.91)	741.6 ± 296.5 (500/100–1200)	< 0.001*

SD, standard deviation

^a^ p-value of t test on the logarithm of health expenditure

*Test significant

Hospitalization was the item with highest amount of expenditure, while drugs were the item with the highest frequency regardless of the place of residence. OOP hospitalization expenses in Abidjan were 2.3 and 2.2 times higher than in rural and urban areas. Followed by OOP expenditures on laboratory/radiology except in rural areas where the amount of OOP expenditure on drugs was more than that of laboratory/radiology. Comparing the three areas of residence, health expenditures from the orthodox medical system was higher in Abidjan; while average spending on traditional medicines in rural and urban areas was 8 times more than that in Abidjan.

Table 2 details the univariate sequential analysis of OOP and the area of residence. In all three areas of residence, age, marital status, quintiles of expenditure, living conditions characterized by the convenience score and the existence of a chronic illness were statistically associated with OOP health expenditure. Expenditure increased with age, with the age group of 5–14 years having the lowest spending. There was less expenditure among single people in all areas of residence. Direct payments were higher in the richest quintile 5 than in the poorest quintile (Q1) in all areas of residence; Abidjan 13,300 XOF (23.74 USD) in Q1 versus 28,950.7 XOF (51.67 USD) in Q5, urban area 8327.5 XOF (14.86 USD) versus 22,292.1 XOF (39.79 USD) and rural area 10,048 XOF (17.93 USD) versus 22,410 XOF (40 USD). The OOP health expenditure increased from the poor living conditions to the better living condition [mean for score 0: rural = 13,600 XOF (24.27 USD) and urban = 13,518.6 XOF (24.13 USD), mean for score 3: rural = 14,428.9 XOF (25.75 USD), mean for score 4: urban = 32,741.7 XOF (58.44 USD)], except in Abidjan where a higher value observed for score 0 was not statistically different. Sex, level of education and household size did not influence OOP health expenditure. By comparing the three areas of residence, OOP health expenditure was highest in Abidjan in most of the variables e.g. sex, age, marital status, household size, insurance or financial aid for treatment expenses (Additional file 1).

**Table 2:** Estimate value of direct payments (XOF) by variables according to place of residence

Variables	Rural (n = 3311)	Urban (n = 2296)	Abidjan (n = 708)	p^a^
	Mean ± SD (Médiane)	Mean ±SD (Médiane)	Mean ± SD (Médiane)	
Gender				
Male	15,060.8 ± 27,215.43 (10,000)	16,830.7 ± 37,946.1 (10,000)	23,334.2 ± 60,489.3 (11,000)	0.003*
Female	13,777.6 ± 23,414.6 (10,000)	15,246.4 ± 27,392.4 (10,000)	23,555.6 ± 49,602.0 (11,000)	0.000*
p	0.7880	0.2837	0.1266	
Age in years				
0–4	10,371.0 ± 12,763.1 (7000)	10,784.7 ± 15,250.0 (8000)	14,385.1 ± 27,398.5 (10,000)	0.0035*
5–14	9273.7 ± 10,062.9 (6500)	9696.1 ± 9143.4 (7000)	11,452.9 ± 11,447.1 (8350)	0.0552
15–34	14,295.3 ± 17,971.1 (10,000)	14,107.8 ± 14,766.3 (10,000)	22,364.4 ± 44,930.0 (10,763)	0.0038*
35–64	17,397.5 ± 31,221.6 (11,000)	21,287.2 ± 47,047.3 (12,000)	31,655.7 ± 38,661.5 (13,000)	0.0065*
≥ 65	23,450.2 ± 54,012.1 (11,000)	30,032.3 ± 70,913.4 (17,500)	31,482.4 ± 38,661.5 (15,250)	0.0029*
p	< 0.001*	< 0.001*	< 0.001*	
Marital status				
Married (e) or Free relationship	16,822.0 ± 29,086.1 (10,400)	19,179.9 ± 38,635.8 (12,000)	29,595.8 ± 72,907.6 (13,000)	< 0.001*
Divorced (e) or widow (er)	15,819.4 ± 17,249.8 (10,400)	25,231.6 ± 61,897.1 (15,000)	26,523.3 ± 34,526 (14,000)	0.0016*
Single	11,290.9 ± 21,002.1 (7250)	11,553.2 ± 15,121.4 (8000)	16,677.1 ± 29,712.2 (10,000)	< 0.001*
p	< 0.001*	< 0.001*	< 0.001*	
Level of education				
Not educated	15,275.2 ± 27,857.45 (10,000)	16,739.1 ± 35,342.6 (10,000)	22,098.4 ± 45,135.9 (12,000)	< 0.001*
Primary	13,902.6 ± 16,782.9 (9750)	13,975.0 ± 18,162.2 (9625)	24,370.4 ± 72,783.0 (10,000)	0.1074
Secondary	17,940.7 ± 39,103.8 (10,500)	18,388.4 ± 45,466.0 (10,000)	21,748.7 ± 30,031.9 (11,000)	0.0950
Superior	14,069.2 ± 16,074.3 (9500)	18,590.7 ± 17,833.7 (13,350)	39,365.2 ± 95,930.9 (10,000)	0.1280
p	0.0933	< 0.001*	0.2696	
Quintile of household’s expenditure on consumables				
1	10,048.2 ± 10,835.5 (7000)	8,327.5 ± 8234.7 (5200)	13,300.0 ± 6184.9 (14,500)	0.0116*
2	10,321.5 ± 10,163.5 (7500)	11,910.4 ± 12,554.5 (8000)	16,322.8 ± 18,991.3 (11,000)	0.0673
3	12,793.5 ± 14,357.1 (10,000)	13,890.0 ± 16,083.1 (9000)	15,919.8 ± 22,026.0 (11,000)	0.0993
4	15,652.7 ± 22,126.5 (10,000)	15,090.7 ± 17,238.0 (10,325)	15,651.1 ± 22,594.0 (10,000)	0.3439
5	22,410.0 ± 46,277.6 (12,000)	22,292.1 ± 54,438.7 (11,500)	28,950.7 ± 69,385.9 (11,000)	0.4711
p	< 0.001*	< 0.001*	0.0277*	
Convenience score				
0	13,600.0 ± 21,423.9 (9300)	13,518.6 ± 27,361.4 (8150)	18,250.0 ± 12,586.4 (17,250)	0.3644
1	15,640.9 ± 32,767.5 (10,000)	13,836.4 ± 21,177.7 (9030)	29,842.0 ± 51,142.1 (10,500)	0.4920
2	15,404.6 ± 17,823.0 (10,000)	17,554.6 ± 43,740.7 (10,500)	22,407.9 ± 62,785.7 (10,500)	0.1333
3	14,428.9 ± 16,637.3 (10,000)	20,824.2 ± 31,873.6 (13,500)	23,368.8 ± 51,978.8 (11,000)	0.4175
4	–	17,230.8 ± 13,450.7 (15,000)	32,741.7 ± 54,566.2 (10,000)	0.2096
p	0.0467*	< 0.001*	0.9131	
Size of households				
1	17,361.3 ± 39,695.6 (10,000)	16,336.0 ± 28,150.5 (9000)	21,111.2 ± 30,747.8 (10,250)	0.6091
2 à 3	15,960.9 ± 30,304.6 (10,000)	15,201.7 ± 22,642.5 (10,000)	24,168.7 ± 37,318.2 (12,000)	< 0.001*
4 à 5	13,713.1 ± 21,595.4 (9230)	17,184.4 ± 46,486.7 (10,000)	24,692.7 ± 74,258.8 (10,000)	0.0057*
6 à 7	12,706.7 ± 19,220.0 (8500)	16,011.3 ± 32,073.8 (10,000)	24,754.4 ± 62,636.6 (11,000)	< 0.001*
> 7	13,271.1 ± 15,119.4 (10,000)	15,075 ± 17,873.5 (10,100)	18,154.6 ± 24,731.1 (11,000)	0.0472*
p	0.1841	0.6702	0.3781	
Chronic disease				
Declared	23,678.9 ± 44,549.4 (14,500)	29,196.2 ± 62,923.0 (15,000)	35,223.5 ± 86,272.5 (13,000)	< 0.001*
Not declared	13,746.1 ± 23,231.0 (9230)	14,788.7 ± 27,017.3 (10,000)	21,923.4 ± 49,618.7 (10,500)	0.9328
p	< 0.001*	< 0.001*	0.0145*	
Insurance or financial aid for medical expenses				
Yes	15,610.2 ± 40,299.0 (8000)	22,352.0 ± 60,048.6 (11,318)	37,317.6 ± 89,391.4 (11,000)	< 0.001*
No	14,345.7 ± 23,738.5 (10,000)	15,172.8 ± 27,318.5 (10,000)	21,376.2 ± 47,743.1 (11,000)	< 0.001*
p	0.1028	0.0010*	0.2423	

^a^p-value of the log of health expenditure

Multivariate analysis (Table 3) indicated that gender, marital status, quintiles of expenditure, being included in a large household size and being insured or receiving financial aid were significant predictors of OOP health expenditure. Compared to the capital Abidjan, living in rural or urban area did not influence direct payment. There was no statistically significant difference between the OOP expenditures of individuals living in Abidjan and other areas of residence (Urban: p = 0.91, rural: p = 0.74). About demographic factors, women spent 26% more than men (p = 0.015). People from large households (more than 7 individuals) spent more than other households (p < 0.001). Age was not a predictor of direct payments. Concerning socio-economic parameters, single people spent 52% less and widowers spent 18% less compared to married or free relationship. Being included in quintiles Q1, Q2, Q3, Q4 decreased direct payments of 133%, 98%, 61% and 35% respectively compared to the richest quintile Q5 (p < 0.001). Living conditions and education level were not statistically associated with direct payments. Concerning assurance and health, being insured or receiving financial assistance reduced direct payments by 69% (p = 0.001). The existence of a chronic disease was not a predictor of OOP expenditures.

**Table 3:** Direct payment determinant: generalized log linear model

Explanatory variables	Global			
	Coeff	SE	p value^a^	CI_95%_
Gender (ref: female)				
Male	0.26	0.11	0.015*	*0.05*; *0.48*
Age in year (ref: > 65)				
0–4	0.41	0.28	0.148	− 0.14; 0.96
5–14	0.07	0.22	0.751	− 0.36; 0.50
15–34	0.10	0.16	0.541	− 0.21; 0.40
35–64	0.06	0.10	0.556	− 0.14; 0.25
Marital status (ref: married)				
Divorced or widowed	− 0.18	0.07	0.015*	*− 0.32*; *− 0.03*
Single	− 0.52	0.10	< 0.001*	*− 0.72*; *− 0.33*
Level of education (ref: superior)				
Not educated	− 0.05	0.08	0.497	− 0.21; 0.10
Primary	− 0.07	0.08	0.417	− 0.23; 0.09
Secondary	− 0.04	0.08	0.617	− 0.19; 0.12
Household size (ref: > 7)				
1	− 1.48	0.20	< 0.001*	*− 1.89*; *− 1.08*
*–3*	− 0.93	0.13	< 0.001*	*− 1.20*; *− 0.67*
4–5	− 0.55	0.08	< 0.001*	*− 0.72*; *− 0.39*
6–7	− 0.28	0.06	< 0.001*	*− 0.39*; *− 0.16*
Quintile of household expenses (ref: 5)				
1	− 1.33	0.20	< 0.001*	*− 1.72*; *− 0.94*
2	− 0.98	0.14	< 0.001*	*− 1.26*; *− 0.71*
3	− 0.61	0.09	< 0.001*	*− 0.79*; *− 0.44*
4	− 0.35	0.05	< 0.001*	*− 0.46*; *− 0.24*
Convenience score (ref: 0)				
1	− 0.02	0.04	*0.65*	*− 0.09*; *0.06*
2	0.06	0.05	0.204	− 0.03; 0.15
3	0.03	0.07	0.655	− 0.10; 0.16
4	0.04	0.16	0.799	− 0.28; 0.36
Area of residence (ref: Abidjan)				
Urban	− 0.01	0.06	0.91	− 0.12; 0.10
Rural	0.02	0.06	0.74	− 0.10; 0.15
Chronic disease (ref: no reported) Chronic disease reported	− 0.15	0.29	0.601	− 0.71; 0.41
Insurance or financial aid for treatment payment (ref: no) Insured or financial aid	− 0.69	0.20	0.001*	*− 1.09*; *− 0.29*
Chronic disease + insured or financial aid	0.32	0.14	0.020*	*0.05: 0.58*
Chronic disease + age in year	0.17	0.06	0.004*	*0.05*; *0.29*
Chronic disease + gender	− 0.20	0.09	0.036*	*− 0.38*; *− 0.01*
Quintile + household size	− 0.06	0.01	< 0.001*	*− 0.08*; *− 0.03*
Quintile + marital status	0.04	0.01	0.001*	*0.02*; *0.06*
Quintile + insured or financial aid	0.10	0.04	0.005*	*0.03*; *0.18*
Total observation	5396			

*Ref* reference, *Coef*. coefficient, *SE* standard error, *CI*_*95%*_ confidence interval

^a^ p-value of the log of health expenditure

### Discussion

Hospitalization expenses are the first item in terms of amount as reported in the literature [23]. Drug expenditures are generally more frequent as observed in Tajikistan [24] and hold the first position in health expenditure [25]. Laboratory expenditures are higher than drug expenditures, contrary to what is usually observed. This opposite situation observed in our study could be explained by the fact that in Côte d'Ivoire, the laboratory/radiology service providers are rather private. Even in the public hospitals, most biological laboratories are run by a private provider or are almost non-functional [26].

The age group of over 65 years spent more as found in most studies [27, 28]. In Côte d'Ivoire, this population is growing and its proportion in the total population has increased from 2.75% in 1998 to 2.89% in 2014 with a projection of 2.92% in 2020 [29]. Elderly people are a fragile population and they are of increasing interest to the research community [30, 31]. They are the most likely to experience chronic diseases that significantly increase health care expenditure [32, 33]. Thus, our study revealed a 33% to 16% increase in direct payments in the cases of chronic diseases in rural and urban areas. In contrast, the low direct payments observed for children under five could be related to the exemption for payments for service fees, currently being applied in public and social approved health care facilities in Côte d'Ivoire.

Women spent more in adequation with the literature as they used health services more [15]. Age was not a predictor of direct payments in Côte d'Ivoire. However, several studies have shown that individuals over 65 years of age spend more [27, 28] due to the existence of chronic diseases [29, 30]. The elderly are a vulnerable population and are of increasing interest to the research community [31, 32]. In Côte d'Ivoire, there is no financial risk protection system for this population often uninsured segment of the population. The proportion of insured persons was 10% in 2015 and concerned active persons [12]. It could be assumed that the over-65s are more likely to use traditional medicine as observed in the Republic of Tanzania [15]. The implementation of an insurance system for elders is important like there is an increase of the size of this population, from 2.75% in 1998 to 2.89% in 2014 (RGPH 2014) with a projection of 2.92% in 2020 [33]. In addition, one would have expected a difference in direct payments for children aged 0–59 months compared to the elderly since children benefit from a policy of exemption from care costs in public institutions. This suggests the ineffectiveness of this policy. Indeed, several studies have shown gaps in the implementation of exemption policies in most of the Sub-Sahara’ countries [13]. The drugs that often constitute the first item of health expenditure are often out of stock. Economic status is also mentioned in the literature as a positive predictor of direct payments [17, 27, 34, 35]. This suggests that people with a better financial condition spend more on their health. Direct payments are related to the willingness and ability to pay. The rich are more likely to patronize private medical facilities and pay high fees. The higher direct payments in Abidjan revealed inequities in health expenditure. The variable areas of residence tell a lot about the level of socioeconomic development and the availability of health facilities. This suggests better health facilities and higher number of private establishment in Abidjan increasing the utilization of health facilities and therefore health expenditure [34].

Individuals from large household size spent more as observed in the literature [34]. This implies for a better control of health expenditure, to insist on a family planning program because sub-Saharan Africa records the highest synthetic fertility and birth rates [36].

Like other studies [10], having insurance coverage or relying on a third party to pay for its health expenditure was a determining factor for direct payment. Otherwise, it is expected that the insurance does reduce the out of pocket expenditure. However the level of health insurance is low in Côte d’Ivoire, almost 10% [12]. The insurance system concern the private insurance. Universal health coverage has not been implemented in 2015, although a law was passed in 2013. This situation implies that private insurance coverage has a weaker impact on health spending in Côte d'Ivoire. Hence the need to put in place at the national level a system of protection against the financial risk linked to medical treatment.

## Limitations

However, our study has some limitations due to the fact that the data we used were collected for a more general assessment of the standard of living of households, which goes beyond health issues. For example, data on health services utilization and health expenditures had different recall periods, 1 month for the first and 3 months for the second. In addition, the 3-month recall period could introduce a memory bias; however, this bias could have been distributed over the entire sample. Expenditure data may have been minimized to some extent. However, the relationships among the sampled areas of residence could not have been affected. Thus, the use of the data which is representative of the population in Côte d'Ivoire, makes it possible to analyze the structuring of the OOP health expenditure and to bring out factors of variation.

## Additional file

**Additional file 1.** Database including 408 variables on individual socio-demographic and economic characteristics of 47635 people.

## Abbreviations

OOP: out of pocket
NIS: National Institute of Statistics of Cote d’Ivoire
